# Evaluation of the impact of fibromyalgia in disease activity and treatment effect in spondyloarthritis

**DOI:** 10.1186/s13075-016-0943-z

**Published:** 2016-02-09

**Authors:** Natalia Bello, Adrien Etcheto, Caroline Béal, Maxime Dougados, Anna Moltó

**Affiliations:** Rheumatology Department, Cochin Hospital, AP-HP, París, France; Rheumatology Department, Hospital General Universitario Gregorio Marañón, Madrid, Spain; INSERM (U1153): Clinical Epidemiology and Biostatistics, PRES Sorbornne Paris-Cité, Paris, France

**Keywords:** Spondyloarhtritis, Fibromyalgia, TNF alpha blockers treatment

## Abstract

**Background:**

Fibromyalgia (FM) can coexist with Spondyloarthritis (SpA) leading to diagnostic and treatment dilemmas, especially in the presence of enthesitis. With this study we aimed to estimate the prevalence of FM in SpA and to compare the clinical/disease features and TNF inhibitors (TNFi) in patients with/without FM.

**Method:**

FM was defined by a score = > 5/6 of the Fibromyalgia Rapid Screening Tool (FiRST). SpA patients (according to the rheumatologist) and consecutively consulting in the day care hospital but also in the outpatient clinic at the rheumatology department of a tertiary care university hospital were included.

Demographics, disease characteristics, activity and severity and TNFi treatment were compared in patients with and without FM; retention rate of the first TNFi and associated factors were explored (Kaplan Meier and Cox regression).

**Results:**

Of the 196 enrolled SpA patients, 42 (21.4 %) were positively screened for FM. No statistically significant differences in the prevalence of FM were found with regard to the fulfillment of the ASAS criteria for peripheral/axial SpA, nor with regard to the fulfillment of the imaging vs. clinical arm of the ASAS criteria. However, patients with coexisting FM presented significantly with more enthesitis, higher disease activity (BASDAI and VAS) and poorer function scores (BASFI). No differences were found with regard to the initiation of TNFi treatment (79.0 % vs. 70.0 %, respectively), but the retention rate of the first TNFi after 2 years was shorter in the group of patients with FM (28.1 % (95 % CI 12.5-44.0) vs. 41.7 % (95 % CI 32.2-51.3), p = 0.01).

**Conclusion:**

This study confirms that coexistent FM in SpA might impact the patient-reported outcome indices for disease activity and function, and the retention rate of TNFi treatment.

## Background

Spondyloarthritis (SpA) is a disease that includes a spectrum of chronic inflammatory entities involving the axial skeleton (sacroiliac joints and spine) and the peripheral joints, and sharing a number of clinical features such as arthritis, enthesitis, uveitis, dactylitis, psoriasis, and inflammatory bowel disease with a common genetic background, the human leukocyte antigen (HLA) B27 [1–4]. In 2009, the Assessment of the Spondyloarthritis International Society (ASAS) proposed a new set of classification criteria with the aim of recognizing patients with early axial SpA (axSpA) including for the first time the imaging of the sacroiliac joints (SIJ) by magnetic resonance imaging (MRI) and abnormal C-reactive protein (CRP) [5], and another set for SpA patients with predominantly peripheral manifestations (e.g., peripheral arthritis, enthesitis, and dactylitis) [6, 7]. The axSpA criteria allow the classification of patients with chronic back pain lasting for ≥3 months and occurring before 45 years of age, through two arms: the imaging arm in which patients present with sacroiliitis (either on radiographs or MRI) plus at least one additional SpA feature, and the clinical arm in which patients need to be HLA-B27 positive and present with two or more SpA characteristics.

However, the clinical arm has been criticized and is not well-recognized by our health authorities nor some colleagues, despite the fact that it has been fully validated [8]. The main argument for such criticism is that this arm would allow the classification of SpA in patients without any objective sign of inflammation (abnormal CRP or presence of inflammatory lesions seen on MRI of the SIJ) or structural damage in the SIJ seen on pelvic radiographs [6, 9].

Indeed, although the ASAS criteria have been proven to be highly specific with acceptable sensitivity both for diagnosis and classifying SpA patients [10, 11], one could consider it inappropriate to apply them as a diagnostic tool in the absence of objective signs of structural damage or inflammation, due to the potential risk of misdiagnosing or over-diagnosing SpA in patients with other conditions (e.g., fibromyalgia (FM), especially in the presence of enthesitis [12].

FM is a complex chronic condition of unknown etiology being considered as a pain amplification syndrome associated with a central nervous system sensitization mechanism [13]. Its hallmark symptoms are chronic widespread musculoskeletal pain and generalized tender points, but other symptoms can be present, such as fatigue, sleep alterations and stiffness leading to significant physical disability and reduced quality of life [14, 15]. Its prevalence has been estimated at around 2–7 % of the general global population and is predominantly found in women [13]. FM can frequently be associated with other rheumatic diseases such as rheumatoid arthritis and Sjögren syndrome [16], and some studies have reported the prevalence of FM at 12.6–15.0 % in SpA, but studies are sparse [16–18]. This FM-SpA association may present diagnostic and treatment dilemmas because some SpA symptoms (e.g., pain at entheses, fatigue, stiffness, and tenderness) can be also found in FM [12, 19]. Few data are available to date, but it has already been reported that SpA patients with FM tend to present with higher self-reported disease activity indices (i.e., Bath Ankylosing Spondylitis Disease Activity Index (BASDAI)) making interpretation of disease activity and treatment response in SpA patients with concomitant FM very challenging [19–21].

In the absence of specific biomarkers for FM, diagnosis can currently be performed using the American College of Rheumatology (ACR) criteria (1990 ACR classification of FM and the ACR 2010 and modified 2010 diagnostic criteria (2011)) [14, 22, 23]. However, these criteria were developed for research and classification purposes, and are difficult to apply in daily practice because they are time consuming and require some training to be implemented [24]. Moreover, such criteria (i.e., ACR 1990) integrate tender points on physical examination that might reflect enthesitis in patients with SpA. Therefore, with the objective of identifying an easy and valid screening tool to facilitate the identification of FM patients in clinical practice and research, the self-reported Fibromyalgia Rapid Screening Tool (FiRST) [25] was developed, which has sensitivity of 90.5 % and specificity of 85.7 % [25] for the identification of FM patients.

All the above prompted us to conduct this study aiming to 1) estimate the prevalence of FM according to the FiRST in a population of patients with SpA and to compare the prevalence with regard to the arm fulfilled within the axial criteria (i.e., the imaging and clinical arms), and 2) compare the demographics/disease features, and TNF inhibitor (TNFi) treatment in terms of initiation and first TNFi retention rate, in patients with and without FM, respectively.

## Methods

### Study design

The completion of the FiRST, BASDAI and Bath Ankylosing Spondylitis Functional Index (BASFI) questionnaires were performed prospectively, and data on demographics and disease features were retrospectively retrieved from the medical files.

### Study population

Patients aged ≥18 years were included, who were diagnosed with SpA (according to the rheumatologist) and were consecutively attending the day care hospital, but also the outpatient clinic at the rheumatology department of a tertiary care university hospital.

### Data collection

FM was defined if the score was ≥5/6 in the FIRST questionnaire [25]. Medical files of each patient who completed the FiRST questionnaire were reviewed by two external investigators. The following information was collected: age, gender, smoking status (past, current, never), body weight and height, and the date of SpA diagnosis, all available items permitting the calculation of the fulfilment of the ASAS criteria; BASDAI [26], global visual analog scale (VAS) according to the patient, presence and number of current swollen joints diagnosed by a physician and CRP at the day of the visit were collected, and severity of the disease evaluated at the time of the visit using the BASFI [27]; SpA treatments since disease onset including information on non-steroidal anti-inflammatory drugs (NSAIDs), conventional disease-modifying antirheumatic drugs (cDMARD), and the type and number of TNFi, number of switches, start/end date and reason for discontinuation for each TNFi. Information on past or current use of psychotropic medications (i.e., myorelaxants, antidepressants, or anxiolytics), strong opioids [28, 29], and history of depression were collected. No imaging (i.e., ultrasound or MRI) was performed specifically for this study.

### Ethics considerations

This study was not submitted to any ethical committee because it was performed using data collected in routine care. However, all patients gave their oral consent to use their data for this present study (as stated by French national official procedures for non-interventional studies).

### Missing data handling

In the case of missing information patients were contacted by telephone to obtain such information. If CRP was not available on the day of the visit, the last available CRP measurement collected within the previous 6 months was used. Furthermore, if any of the answers to the FiRST questionnaire were missing, we only excluded the patients for whom the missing answers did not allow us to determine their group (with/without FM), e.g., a patient who positively answered three questions but did not answer the last three questions.

### Statistical analysis

Continuous variables were reported by their mean and standard deviation (SD) and qualitative variables by frequency and percentage. Statistical significance was set at *p* <0.05. The analysis was performed with the statistical software SAS 9.4.

Evaluation of the reliability of the FiRST questionnaire was performed. It was evaluated in a subset of 22 patients in two consecutive visits. These patients had stable disease (∆BASDAI between two visits: 0.22 (±1.32)) and no treatment changes. The average time between the two visits was 22 weeks (± 7.68). Reliability of the FM diagnosis according to the FiRST definition was assessed by prevalence-adjusted bias-adjusted kappa statistics (PABAK).

FM prevalence was estimated in the global SpA population, but also with regard to the ASAS classification criteria fulfilment (axial or peripheral) and to the fulfilment of the imaging vs. clinical arm of the ASAS criteria for axSpA. Demographics, disease characteristics, activity and severity were compared in the FM+/FM– groups by the *t* test and chi square (χ^2^) test, as appropriate.

The percentage of patients who were ever exposed to a TNFi, the mean number of TNFi received, the mean duration of the first TNFi treatment and the reasons for discontinuation of each TNFi were assessed in the total population and compared in the FM+ /FM– groups. The retention rate of the first TNFi treatment in the FM+/FM– groups was estimated by survival analysis (Kaplan–Meier curves) and compared by the log-rank test. The predisposing factors for discontinuation of the first TNFi during the first 2 years were estimated by Cox regression models first by univariate and thereafter by multivariate analysis, including in the model only the variables that had a *p* value <0.10 in the univariate analysis, plus age and gender. Finally, the percentage of patients who received ≥3 TNFi within 12 months (*switchers*) in both groups was compared by the χ^2^ test.

## Results

Of the 213 patients who completed the FiRST questionnaire, 196 were retained for our analysis: patients without a confirmed diagnosis of SpA (n = 14) and patients who incompletely answered the questionnaire (n = 3) were excluded. The reliability of the FM diagnosis was good, with a PABAK = 0.64 (95 % CI 0.314; 0.958). In our SpA population, the prevalence of concomitant FM was 21.4 % (42/196).

### Fibromyalgia prevalence according to the ASAS classification criteria

**Fig. 1:**
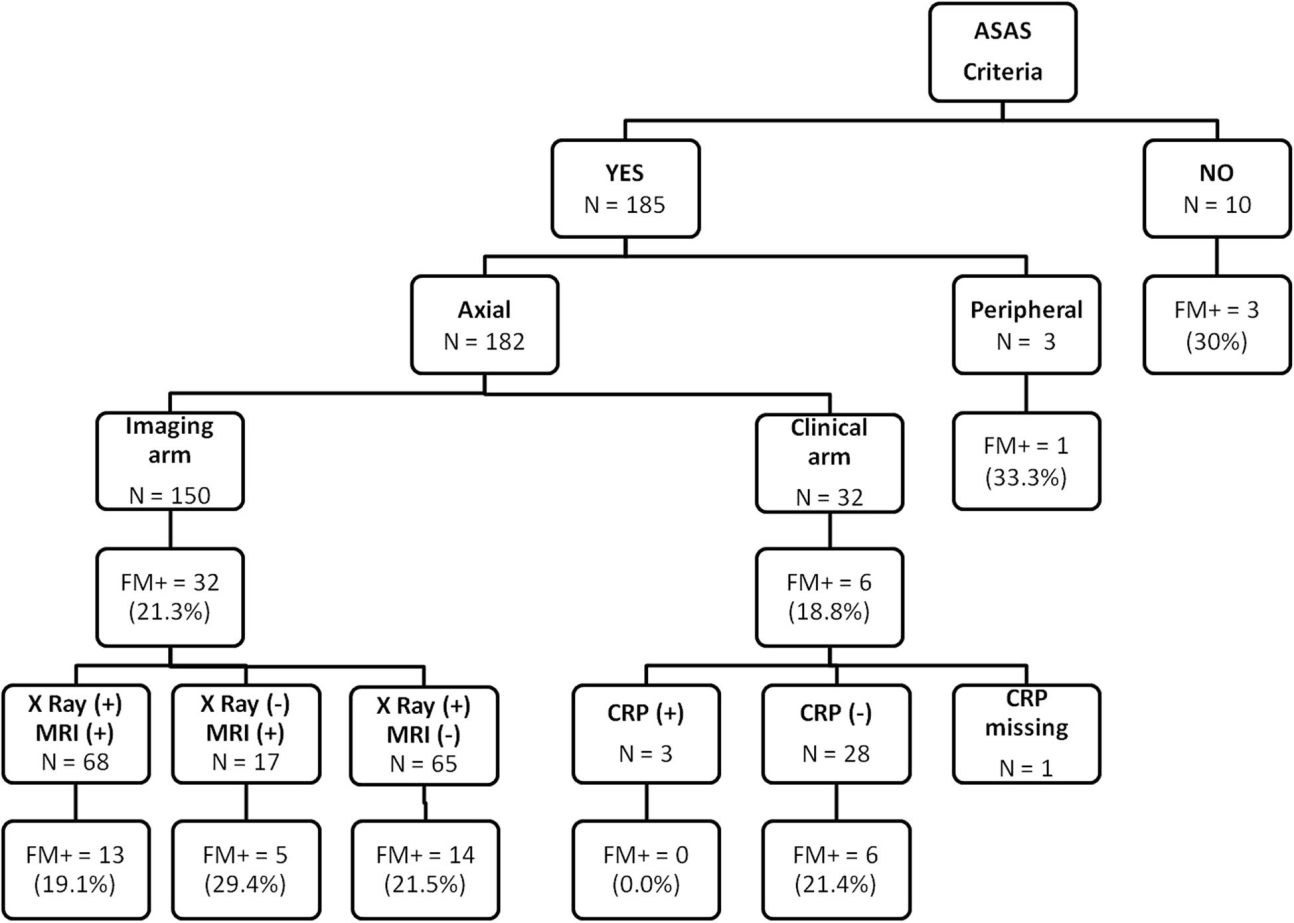
Fibromyalgia (*FM*) in spondyloarthritis depending on the Assessment of the Spondyloarthritis International Society (*ASAS*) classification criteria and the arm fulfilled. *FM+* fibromyalgia defined by the Fibromyalgia Rapid Screening Tool (*FIRST*) questionnaire (i.e., FIRST ≥5/6), *XRay(+)* radiographic sacroiliitis, *MRI(+)* magnetic resonance imaging sacroiliitis, *CRP(+)* abnormal C-reactive protein (i.e., ≥6 mg/L)

Figure 1 summarizes the prevalence of FM depending on the ASAS classification criteria. Of the 196 patients included in the analysis, 185 (94.4 %) met the ASAS criteria (182 (98.4 %) and 3 (1.6 %) axial and peripheral, respectively). For the ASAS criteria for axSpA, 150 patients (82.4 %) and 32 patients (17.6 %) fulfilled the imaging and clinical arms, respectively.

Prevalence of concomitant FM was greater in the group of patients not fulfilling the ASAS criteria, although this difference was not statistically significant (21.1 % vs. 30.0 %, not significant). More interestingly, no differences in the prevalence of FM were observed in the group of patients fulfilling the imaging and clinical arms of the ASAS criteria for axSpA (21.3 % vs. 19 %, not significant).

Demographics, disease characteristics, activity and severity were compared in the FM+ and FM– groups (see Table 1). These two groups were similar in terms of age, mean age at disease onset and smoking status. However, patients fulfilling the FM+ definition presented more frequently with enthesitis (59.5 % vs. 39.0 %, *p* = 0.01), a higher total BASDAI (4.7 (±2.3) vs. 2.6 (±1.9), *p* <0.01), higher global VAS (5.9 (±2.4) vs. 3.0 (±2.5), *p* <0.01) and higher BASFI (4.8 (±2.7) vs. 2.0 (±2.3), *p* <0.01). No significant differences were found for treatment with non-steroidal anti-inflammatory drugs (NSAIDs) and conventional disease-modifying antirheumatic drugs (cDMARDs); as expected, the percentage of patients with either history of depression, or use of psychotropic medication or strong opioids was significantly higher in the FM+ group (67 % vs. 35 %, *p* <0.01).

**Table 1:** Demographic and disease characteristics of patients with and without fibromyalgia

	Total number of patients	Fibromyalgia+	Fibromyalgia–	*P*
	(n = 196)	(n = 42)	(n = 154)	
Age, years	43.0 (12.6)	41.4 (10.2)	43.4 (13.13)	0.21
Gender, female	59 (30.1 %)	17 (40.5 %)	42 (27.3 %)	0.10
Age at disease onset, years	29.9 (11.9)	28.7 (9.3)	30.2 (12.5)	0.40
Smoking status, ever smoked	106 (55.8 %)	23 (56.1 %)	83 (55.7 %)	0.96
HLA B27-positive	157 (82.2 %)	35 (83.3 %)	122 (81.9 %)	0.83
Radiographic sacroiliitis	134 (70.2 %)	27 (67.5 %)	107 (70.9 %)	0.68
Magnetic resonance imaging evidence of inflammatory lesions of the sacroiliac joints	88 (58.3 %)	20 (54.1 %)	68 (59.7 %)	0.55
Raised C-reactive protein*	39 (22.3 %)	8 (20.0 %)	31 (23.0 %)	0.69
Past history of, or current arthritis	72 (36.7 %)	12 (28.6 %)	60(39 %)	0.22
Past history of, or current enthesitis	85 (43.4 %)	25 (59.5 %)	60 (39 %)	0.01
Bath Ankylosing Spondylitis Disease Activity Index total (0–10)	3.0 (2.2)	4.7 (2.3)	2.6 (1.9)	<0.01
Global visual analog scale (0–10)	3.6 (2.8)	5.9 (2.4)	3.0 (2.5)	<0.01
Bath Ankylosing Spondylitis Functional Index (range 0–10)	2.6 (2.7)	4.8 (2.7)	2.0 (2.3)	<0.01
History of depression	42 (23.2 %)	16 (41.0 %)	26 (18.3 %)	<0.01
Past or current intake of psychotropic medications** or strong opioids	82 (41.8 %)	28 (66.7 %)	54 (35.1 %)	<0.01
Past or current intake of psychotropic medications or strong opioids or history of depression	82 (41.8 %)	28 (66.7 %)	54 (35.1 %)	<0.01
Non-steroidal anti-inflammatory drug intake, ever	193 (99.0 %)	42 (100.0 %)	151 (98.7 %)	1.00
Synthetic disease-modifying antirheumatic drug use, ever	104 (53.1 %)	23 (54.8 %)	81 (52.6 %)	0.80

All results are presented as mean ± SD for continuous variables or number (%) for categorical variables. Fibromyalgia + was defined as a Fibromyalgia Rapid Screening Tool (FiRST) score ≥5/6. * ≥ 6 mg/L; **myorelaxants, antidepressants or anxiolytics

### TNFi treatment

Table 2 summarizes TNFi treatment characteristics in our population. The percentage of patients ever exposed to TNFi did not differ between the FM+ vs. FM– groups (79 % vs. 70 %, respectively, not significant), whereas the percentage of switchers was significantly higher in the FM+ group (15.2 % vs. 4.0 %, *p* = 0.03). Mean duration of the first TNFi was significantly shorter in the FM+ group (1.7 (± 2.4) vs. 3.5 (± 4.0) years, *p* <0.01) (Table 2).

**Table 2:** TNF inhibitor (TNFi) treatment in patients with and without fibromyalgia

	Total number of patients	Fibromyalgia+	Fibromyalgia-	*P*
	(n = 196)	(n = 42)	(n = 154)	
Patients who received at least one TNFi	141 (71.9 %)	33 (78.6 %)	108 (70.1 %)	0.28
Number of TNFi treatments	1.84 (1.0)	2.36 (1.1)	1.68 (0.9)	<0.01
Switchers*	9 (6.5 %)	5 (15.2 %)	4 (3.8 %)	0.03
First TNFi				
Mean duration of first TNFi treatment, years	3.0 (3.8)	1.7 (2.4)	3.5 (4.0)	<0.01
Reason for TNFi discontinuation:				
Inefficacy %	54 (69.2 %)	12 (54.6 %)	42 (75.0 %)	0.14
Toxicity %	4 (5.1 %)	2 (9.1 %)	2 (3.6 %)	0.76
Inefficacy + toxicity %	4 (5.1 %)	3 (13.6 %)	1 (1.8 %)	0.23
Others %	16 (20.5 %)	5 (22.7 %)	11 (19.6 %)	1.00

All results are presented as mean ± SD for continuous variables or number (%) for categorical variables. Fibromyalgia + was defined as a Fibromyalgia Rapid Screening Tool (FiRST) score ≥5/6. *Patients who received ≥3 TNFi within ≤12 months

**Fig. 2:**
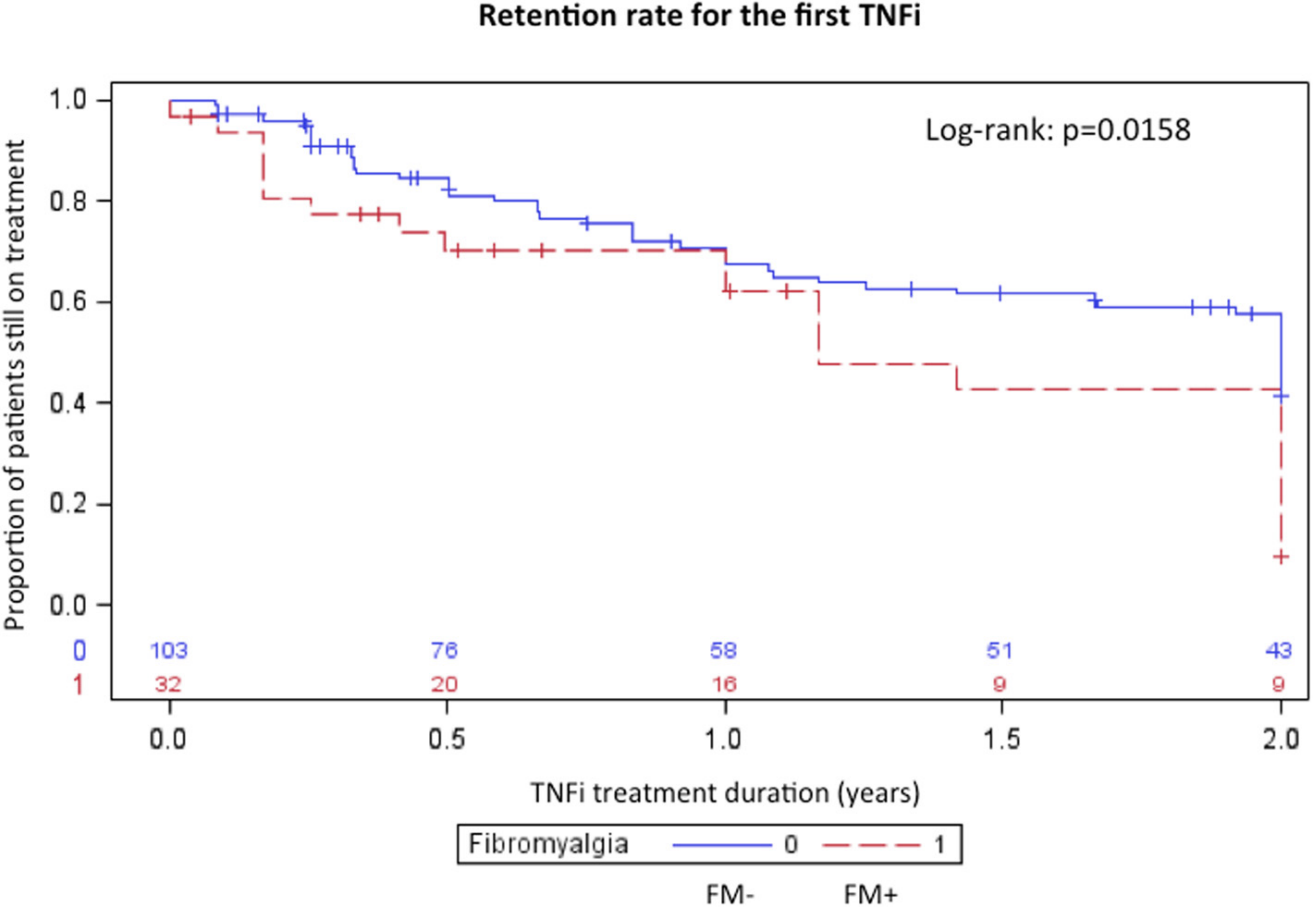
Kaplan–Meier curve for retention rate of first TNF inhibitor (TNFi) during the first 2 years. *FM* fibromyalgia

The retention rate of the first TNFi after 2 years was significantly shorter in the FM+ group: 28.1 % (95 % CI 12.5; 44.0) (n = 9) vs. 41.7 % (95 % CI 32.2; 51.3) (n = 43) of patients were still on TNFi treatment after 2 years in the FM+ and FM– groups, respectively (*p* <0.01) (Fig. 2).

Univariate Cox analysis identified FM (hazard ratio (HR) 1.8, 95 % CI 1.1; 3.0), peripheral involvement (HR 1.6, 95 % CI 1.0; 2.6) and history of depression or psychotropic medications or strong opioids intake (HR 0.6, 95 % CI 0.4; 0.9) as associated factors for discontinuation of the first TNFi; however, on multivariate analysis only FM (HR 1.7, 95 % CI 1.0; 2.9) and peripheral involvement (HR 1.6, 95 % CI 1.0; 2.6) were independently associated with discontinuation of the first TNFi. Reasons for discontinuation of each TNFi were similar percentages in the two groups (*p* value not significant), inefficacy being the most frequent reason in the total population.

## Discussion

To our best knowledge, this is the first study aiming to evaluate the prevalence of FM in a population of patients with SpA with regard to the fulfilment of the ASAS classification criteria, and our results show that no differences were found between the imaging and clinical arms of the ASAS criteria for axSpA. This finding is an argument in favor of the validity of such criteria, particularly for the clinical arm, which is still not fully accepted by some members of the scientific community. The phenotype of SpA patients with concomitant FM was more frequently female and presented more frequently with enthesitis, reporting greater disease activity and poorer function scores. These results are consistent with previous studies [16, 17, 20, 30, 31].

As SpA patients with coexisting FM frequently present with a higher disease activity score, the evaluation of disease activity and treatment effect might be challenging, and might lead to unnecessary initiation of a TNFi, dose escalations or switches. In our study, the percentage of patients initiating a TNFi did not differ in the FM+ and FM– group, but patients with concomitant FM+ disease were more likely to switch to other TNFi treatments. Moreover, the retention rate of the first TNFi was shorter in the FM+ group and the presence of FM was independently associated with a first TNFi discontinuation. This confirms that the existence of concomitant FM in SpA might complicate the evaluation of treatment response [19, 20], and suggests that coexistence of FM should be carefully screened when initiating a TNFi and/or evaluating its treatment effect, especially in the presence of peripheral and/or enthesitic symptoms and in the presence of very severe disease activity and patient-reported scores. In this sense the FiRST questionnaire represents an easy to use and valid tool that might be used in clinical practice before starting a TNFi, in particular in patients presenting with enthesitis.

Our study has some weaknesses but also some strengths. First the diagnosis of FM according to the rheumatologist was not collected. However, we verified the external validity of the FiRST in this SpA setting by confirming the greater use of psychotropic medication or strong opioids, or the history of depression in the group of patients positively screened as FM+ [32, 33]. Second, lower sensitivity of the FIRST when applied in an SpA population compared to the original study (66 % vs. 90 %) has been recently reported [34]; however, these data were not available at the time the study started. Furthermore we did not use the ACR criteria (1990 ACR classification of FM [14] - modified 2010 preliminary ACR criteria [22]) to validate our results on the prevalence of FM, but this was due to the complexity of applying such criteria in a daily practice setting [23]. Finally, one could consider that the sample size of our study was too small to draw definite conclusions, e.g., in the evaluation of the retention rate of the first TNFi, only nine patients were at risk after 18 months of follow up in the FM+ group.

Nevertheless, our study also has some strengths. First, our analyses were performed on a representative number of SpA patients in daily practice. Furthermore, to the best of our knowledge this is the first study aiming to evaluate the prevalence of FM according to the fulfilment of the ASAS classification criteria and its impact on TNFi treatment.

## Conclusions

In summary, the similar percentages of FM in the different subgroups of the ASAS classification criteria might be a good argument in favor of the validity of these criteria, and in particular of the clinical arm. The coexistence of FM might impact the score of the instrument used to evaluate disease activity and severity, particularly in the patient-reported scores, and therefore might complicate the evaluation of treatment response. The implementation of the FiRST questionnaire might be helpful in clinical practice, especially in the presence of enthesitic symptoms. Other studies aiming to prospectively evaluate the impact of concomitant FM in SpA in the treatment effect of TNFi should allow us to confirm (or not) our findings.

## Abbreviations

ACR: American College of Rheumatology
ASAS: Assessment of Spondyloarthritis International Society
axSpA: axial SpA
BASDAI: Bath Ankylosing Spondylitis Disease Activity Index
BASFI: Bath Ankylosing Spondylitis Functional Index
cDMARD: conventional disease-modifying antirheumatic drug
CRP: C-reactive protein
FiRST: Fibromyalgia Rapid Screening Tool
FM: fibromyalgia
MRI: magnetic resonance imaging
NSAID: non-steroidal anti-inflammatory drug
PABAK: prevalence-adjusted bias-adjusted kappa statistics
SIJ: sacroiliac joint
SpA: spondyloarthritis
TNFi: tumor necrosis factor inhibitor
VAS: visual analog scale
